# Solution Gutta Percha in Cloroform

**Published:** 1853-01

**Authors:** 


*Solution Gutta Percha in Cloroform.* By Prof. L. A. Dugas.

This is made by dropping into a vial containing chloroform small fragments of pure gutta percha, until the solution acquires the consistence of thick mucilage. It is then applied with a camel-hair pencil, which should afterward be repeatedly dipped in pure chloroform and carefully wiped with paper or old linen, so as to prevent its becoming stiff and unfit for further use.

I will now relate the result of its application in two cases of cancerous affection.

Mr. L—— had been troubled with an epithelial-cancer of the lower lip which had resisted all applications for eighteen months. There existed, upon the right side of the median line and at the junction of the skin and mucous surface, a small and thin scale or scab, which would occasionally fall or be rubbed off, leaving a raw surface of exquisite sensibility exposed to irritation until another scab would be formed. Beneath this surface there was an induration about the size of a common pea, or rather a little larger, in which the patient felt a very annoying sense of burning, and sometimes darting pain.

At this stage of the case, as the patient was averse to the knife, he was advised to try the application of collodion, which he diligently persevered in for about six months, applying it three or four times daily. This arrested the farther growth of the disease, relieved its itching and burning, protected its surface from ordinary irritants, but did not heal the denuded surface. He then substituted the solution of gutta percha in chloroform in lieu of the collodion. In a letter to me he thus describes its effects: "In a few days I saw and felt a change in the color of the sore and in the irritation; in a week or ten days, the lump disappeared and the irritation subsided, and in three weeks it was almost entirely healed over; in less than a month it was well, leaving an indentation on the lip."

In a note dated the first of this month, (April,) my patient writes me: "I begin to fear a return of it in the same place the cure was made eighteen months ago. Recently a lump has appeared in the lip; it is *hard* and sometimes a little sore—it gives me no trouble yet, but I am afraid of it." I will advise him to use the gutta percha and chloroform again.

The next case was that of Maria, a negress, about fifty years of age, who was sent to me from the country on the 3d of November last, with a cancerous ulceration of the mamma of several months standing. Both mammary glands were very much atrophied, but the affected one was the smaller of the two, presented nothing but a mass of schirrous induration which seemed adherent to the thoracic walls, and in the depressed center of which the remains of a nipple were to be seen drawn back and ulcerated. The ulcer covered a surface equal to the areola. The axilary glands were much enlarged, and the patient a prey to continual pain, especially at night, which deprived her of sleep.

Feeling satisfied that the knife promised no relief under such circumstances, yet unwilling to send her off without trying something, I put her upon the use of the gutta percha and chloroform, thoroughly coating the whole breast daily with it. The discharge from the ulcers would at first cause the pellicle of gutta percha to become loosened in twenty-four hours, so that the surface had to be cleansed before the reapplication of the remedy. The suppuration, however, gradually lessened until the coating could remain a week — the painting still being made each morning. Under this treatment, the patient was gradually relieved of all pain about the breast and even in the axilla. She slept quietly at night, enjoyed her meals, and felt quite well. Her general health improved and she left at the end of one month, with instructions to continue the treatment perseveringly, and to get her master to inform me of the result. I have had no report from her since, but have learned incidentally that she never applied the remedy after she left here, and placed herself under the charge of some one who professed to be able to cure cancer —with what result, I know not.

These two cases are narrated with the simple purpose of directing attention to an application which may stay, if it does not cure, so formidable an affection as cancer.—*Trans. Med. Society State Georgia*, 1852.

